# On the Treatment of Certain Surgical Affections by Elevation of the Diseased Parts

**Published:** 1847-06

**Authors:** 


*On the Treatment of certain Surgical Affections by Elevation of the Diseased Parts.*—M. Gerdy has for some time been in the habit of treating certain inflammatory affections by placing the limb, or part, in such a position as to favour the return of blood to the heart. This plan has this advantage, that it does not exclude the application of the usual means of treatment; but, as is shown by M. Dupuy, it is in many cases in itself sufficient to effect a cure.

The question meets us *in limine.* What are the phenomena which are induced by elevating a part of the body? If the hand, for instance, be allowed to hang down, we observe that it becomes engorged with blood. Place it in the contrary position, and the livid colour disappears, and the vessels empty themselves. It is evident from this experiment that in the first instance the blood accumulates in the most depending part; in the other the reverse occurs, the blood readily finding its way toward the centre of the circulation. What is thus seen to occur in a healthy condition of parts, also happens under certain modifications in disease. M. Dupuy gives the following account of the practical application of the above principles:—

If the thumb or hand be inflamed, the patient is made to lie in such a position that the elbow is maintained in a position higher than the shoulder. The fore-arm is placed perpendicular, supported by cushions, care being specially taken that the circulation is not impeded by bandages; the hand is then enveloped in bandages, to which tapes are fixed, and attached to the top of the bed. These means, with some simple modifications, are likewise made use of in inflammation of the lower extremities. M. Gerda raises the end of the bed by placing a chair under it, thus raising the foot upon the summit of an inclined plane. Once so placed, and care being taken that no injurious pressure is exerted, the patient must not move from the position even to satisfy natural wants; for he may destroy in a few minutes all the benefits which have been obtained by whole days of repose. Although elevation cannot be so efficaciously applied to the *head* and *trunk* as to the extremities, it yet may be employed to a certain extent. Supposing the *eye* to be inflamed,—the patient will lie with his head high, and on the opposite side to the one affected. Why are inflammatory affections and discharges from the *womb* so tedious in recovery, but for the stagnation of the blood in the organ? Let a woman, who has been accustomed to keep herself in the vertical posture, go to bed, and raise her hips by means of pillows, and she will soon find her case amended. The same principles apply to inflammatory affections of the face, breast, &c.

The advantage of this plan of treatment is not, however, confined to inflammation, but it is equally serviceable in ulcers, uterine hæmorrhages, and varicose veins. In many instances of the latter disease, in M. Gerdy's wards, elevation alone of the limb has been completely successful. The utility of the plan is also incontestable in varicocele. The communication of M. Dupuy terminates with these conclusions:

1st. That elevation of the diseased part is able, without the intervention of other therapeutical measures, to cut short certain inflammations, if it be employed sufficiently early.

2nd. That in phlegmon it relieves pain by diminishing the quantity of blood in the part.

3rd. That it advances the cure of engorgements and chronic profluvia of the uterus.

4th. That certain hæmorrhages may be suspended by it.

5th. That it is able to cure certain ulcers of the lower extremities.

6th. That varices and hæmorrhoids are advantageously modified by elevation.

7th. That where it is not sufficient in itself to effect a cure, it is always a potent auxiliary.—*Provincial Med. and Surg. Journ., from Archives Générales.*

